# Enabling Physicians to Make an Informed Adoption Decision on Artificial Intelligence Applications in Medical Imaging Diagnostics: Qualitative Study

**DOI:** 10.2196/63668

**Published:** 2025-08-12

**Authors:** Jasmin Hennrich, Eileen Doctor, Marc-Fabian Körner, Reeva Lederman, Torsten Eymann

**Affiliations:** 1 University of Bayreuth, Bayreuth, Germany; 2 FIM Research Center, Bayreuth, Germany; 3 Branch Business & Information Systems Engineering of the Fraunhofer FIT, Wittelsbacherring 10, Bayreuth, 95444, Germany, 49 160 20304; 4 School of Computing and Information Systems, The University of Melbourne, Melbourne, Australia; 5 Chair for Information Systems and Digital Society, University of Bayreuth, Bayreuth, Germany

**Keywords:** artificial intelligence, medical imaging diagnostics, adoption, user enablement

## Abstract

**Background:**

Artificial intelligence (AI) applications hold great promise for improving accuracy and efficiency in medical imaging diagnostics. However, despite the expected benefit of AI applications, widespread adoption of the technology is progressing slower than expected due to technological, organizational, and regulatory obstacles, and user-related barriers, with physicians playing a central role in adopting AI applications.

**Objective:**

This study aims to provide guidance on enabling physicians to make an informed adoption decision regarding AI applications by identifying and discussing measures to address key barriers from physicians’ perspectives.

**Methods:**

We used a 2-step qualitative research approach. First, we conducted a structured literature review by screening 865 papers to identify potential enabling measures. Second, we interviewed 14 experts to evaluate the literature-based measures and enriched them.

**Results:**

By analyzing the literature and interview transcripts, we revealed 11 measures, categorized into Enabling Adoption Decision Measures (eg, educating physicians, preparing future physicians, and providing transparency) and Supporting Adoption Measures (eg, implementation guidelines and AI marketplaces). These measures aim to inform physicians’ decisions and support the adoption process.

**Conclusions:**

This study provides a comprehensive overview of measures to enable physicians to make an informed adoption decision on AI applications in medical imaging diagnostics. Thereby, we are the first to give specific recommendations on how to realize the potential of AI applications in medical imaging diagnostics from a user perspective.

## Introduction

### Background

As artificial intelligence (AI) technology continues its relentless march across various sectors, its transformative impact is increasingly evident. From finance, where algorithms improve security [1], to energy, where AI may facilitate the integration of renewable energy sources [2], to health care, where it promises to enhance patient care and reduce physicians’ workload [3]. Looking at different disciplines in the health care sector, radiology is the area that is already most engaged with the adoption of AI applications in practice due to its data-driven nature and the predominantly structured image data available [4]. AI applications are transforming medical imaging diagnostics. For example, AI applications can increase the accuracy of breast cancer screenings [5], reduce the need for invasive biopsies in prostate cancer diagnosis [6], or serve as an effective triage tool for prioritizing patients based on urgency [7], with high expectations regarding the reduction of physicians’ workload [8].

Considering these promised benefits, AI applications are expected to be used widely in imaging diagnosis. However, despite their potential, the practical implementation of AI applications in health care has not progressed as quickly as expected [9]. Implementation in the real world faces significant hurdles that make integration into daily practice difficult. Especially, the health care domain demands strict adherence to regulatory standards, seamless integration into complex clinical workflows, and transparency to maintain trust in clinical decision-making. The stakes are significantly higher in medical settings, where AI directly impacts patient care. This problem is exacerbated by physicians’ general reluctance to adopt new technologies, a trend that has already been observed in studies on health information technology (HIT) adoption [10].

The gap between the recognized potential of AI applications in diagnosis and their adoption in medical practice can mainly be attributed to shortcomings in 4 key areas as recent literature points out: macroeconomic, organizational, technological, and user area [11]. Following Roppelt et al [11] on the macroeconomic level, the regulatory landscape for AI applications in health care is characterized by significant uncertainties regarding liability and compliance with medical standards. On the organizational level, many health care organizations still lack the necessary technical infrastructure to integrate AI applications effectively. In terms of technology, there are challenges such as data sensitivity, algorithm transparency, and integration into existing health care systems. User readiness is influenced by various factors, such as physicians’ expectations [12], concerns about AI application usage [8], and physicians’ knowledge and literacy of AI technology [13].

In information systems (IS) research, there is an entire line of literature that deals with the adoption of technologies from a user perspective [14,15]. Adoption comprises the users’ acceptance of the technology as well as the user adoption decision [15,16]. Previous research has already explored various factors influencing physicians’ acceptance of AI applications in medical diagnosis [8,11,13,17]. These studies reveal AI knowledge and literacy gaps [13] and concerns such as job loss, loss of autonomy, additional effort [8], diagnostic bias, and misuse of data to be hindering adoption factors [12], to name a selection. Once the barriers to user acceptance have been identified, a holistic approach is needed to address them to realize the promised benefits of AI in the specific area of imaging diagnosis [18].

Previous literature presents frameworks to accelerate the adoption of AI applications in the health care context. For example, the study by Gama et al [19] conducts a review to identify frameworks for AI implementation in health care, concluding that further empirical research is necessary to develop AI-specific implementation frameworks. The study by Marco-Ruiz et al [20] provides specific implementation guidelines that are based on interviews with implementers. While these frameworks focus on overarching strategies and technical guidelines for integrating AI into clinical settings, they often lack the specificity required to address user-related challenges, particularly from physicians’ perspectives. Our study bridges this gap by identifying targeted measures that empower physicians to make informed adoption decisions, emphasizing the importance of user-centered approaches.

As technology should not be used for its own sake but rather to support users in their tasks, it is particularly important to enable physicians to make an informed adoption decision regarding AI applications. While existing research has identified key barriers to the adoption of AI applications, there is a lack of concrete, user-centered measures that address these challenges from a physician’s perspective. Without clear guidance, physicians may struggle to navigate the adoption process, leading to hesitancy and suboptimal integration of AI application into clinical workflows. Ensuring that physicians can make informed adoption decisions is crucial for leveraging AI’s full potential to enhance diagnostic accuracy, streamline workflows, and ultimately improve patient outcomes. Therefore, this study aims to address the following research question: which measures enable physicians to make an informed adoption decision on AI applications in medical imaging diagnostics?

To answer this research question, we use a 2-step qualitative research approach. First, we conducted a structured literature review to identify potential measures from existing research. Second, we carried out expert interviews with radiologists and AI specialists to refine and validate these measures, ensuring their relevance and applicability in real-world clinical settings.

### Factors Influencing Physicians' AI Adoption in Medical Diagnoses

Researching technology adoption on an individual level is a core focus of IS research [14,15]. Adoption primarily concerns the individual user’s acceptance of technology as well as the adoption decision [15,16]. To explain technology adoption, the most well-known IS theories from an individual perspective are the Technology Adoption Model (TAM) [21] and the Unified Theory of Acceptance and Use of Technology (UTAUT) [14]. UTAUT has been applied to examine factors influencing physicians’ adoption of intelligent clinical diagnostic decision support systems, the use of health clouds by health care professionals, and electronic medical record system adoption [22-24]. TAM, on the other hand, is often criticized as insufficient for explaining technology adoption in the health care context as it fails to account for the qualitative, emotional, and cultural aspects specific to this field [25,26]. Contextualizing these explanatory approaches is crucial [24,27,28], particularly for the unique characteristics of the health care domain [28,29].

While these models provide valuable theoretical insights, they primarily rely on quantitative approaches that measure predefined factors, often overlooking the nuanced, experience-driven aspects of physicians’ decision-making processes. A qualitative approach allows for a deeper exploration of the concerns, expectations, and contextual influences that shape AI adoption in medical imaging diagnostics, providing richer, more actionable insights for practice and policy.

Consequently, this call for contextualization is met with studies on factors that explain the adoption of AI applications in the context of medical imaging diagnostics [8,12,13]. A recent study by Hua et al [12] aimed to summarize all context-specific factors, which existing literature has already identified empirically. Thereby, they provide a comprehensive overview of factors influencing the adoption of AI applications in medical imaging diagnostics. By conducting a scoping review of health informatics literature, they revealed a total of 12 context-specific factors influencing the adoption of AI applications in medical imaging diagnostics. Table 1 shows an overview of the factors, including their definitions.

The overview of currently known contextual factors influencing the adoption of AI applications in medical image diagnosis allows for an assessment of which measures enable physicians to make an informed adoption decision of AI applications. This, in turn, promotes widespread adoption from the user’s perspective.

**Table 1.** Overview of the factors influencing the adoption of artificial intelligence (AI) applications in medical imaging diagnostics according to Hua et al [12].

Influencing factors	Definition
Burden	Capture a physician’s perception on how easy the AI technology will be to use and how much effort will be required to engage.
Value proposition	Capture a physician’s subjective assessment of the extent to which using AI technology can help improve their own performance or achieve certain goals.
Perceived threat	Captures a physician’s perception of how AI is a threat to their professional role.
Trust	Captures a physician’s belief that AI is safe and dependable to use.
Self-efficacy	Captures a physician’s perception of their ability (eg, availability of skills).
AI literacy	Captures a physician’s general understanding and knowledge concerning AI.
System understanding	Captures a physician’s understanding of the specific AI technology they are using.
Ethicality	Captures a physician’s perception that AI fits their value system (eg, data security).
Workflow integration	Captures a physician’s perception on how the integration of AI will be compatible and preserving with the workflow.
Technology receptiveness	Captures a physician’s willingness to try out new innovations.
Social influence	Captures a physician’s perception of how important it is to fulfill or meet the expectations and opinions of others regarding the use of AI technology.
Organizational readiness	Captures a physician’s belief that their organization has resources available (eg, necessary infrastructure) to use the AI technology.

## Methods

### Data Collection

A 2-stage qualitative research methodology was used to uncover measures to enable physicians to make an informed adoption decision regarding AI applications in medical imaging diagnostics. First, a structured literature review was conducted according to Webster and Watson [30] to systematically review the existing literature for already discussed measures of enablement. Subsequently, interviews were conducted to assess and evaluate the identified measures and to collect additional measures. This 2-phase approach ensures not only a thorough consideration of the relevant existing literature on measures but also in-depth information due to the interviews, which contextualize the found measures [31].

Commencing with the data collection phase grounded in literature, we followed the guidelines laid out by Webster and Watson [30] and started with formulating a search string. Through an iterative process, we refined the search string 5 times, ultimately arriving at the following final search string: (adoption OR implementation) AND “artificial intelligence” AND (solution OR “success factor” OR increase OR improve OR facilitate) AND health.

While our paper primarily focuses on the area of medical imaging diagnostics, we deliberately refrained from narrowing down the search string to this specific area. This decision was driven by our assumption that most of the existing measures will not only count for AI applications in this specific area. For the search, we decided on PubMed, an established database for health care journals, and Science Direct, an established database for IS journals. Our initial search of these databases returned 1019 results. We filtered out books (n=80) and duplicates (n=80), leaving 859 articles for screening. Our primary focus was on identifying measures rather than adoption barriers. We excluded 715 abstracts due to a lack of relevant measures (631 lacked evidence for measures, 49 were unrelated to AI, and 35 were not relevant to health care). The remaining 144 abstracts were categorized into two groups: 92 addressed AI adoption barriers but did not propose concrete measures and were excluded, while 52 demonstrated evidence of specific measures and proceeded to full-text screening. After removing 4 inaccessible articles and excluding 32 postscreening, 16 articles remained. A forward and backward search added 3 more, resulting in a final set of 19 articles forming the basis for our analysis of medical imaging diagnostics.

In the second step, we conducted semistructured expert interviews to evaluate and expand the identified measures. Following Schultze and Avital [31], the use of semistructured expert interviews enabled a focused examination of the research topic and, at the same time, provided in-depth insights into potential measures [32]. The questions were designed to allow open-ended discussions while ensuring that all key topics were systematically covered. The interview guide was developed based on existing literature and expert discussions within our research team to ensure relevance. To ensure a common understanding of AI, we clarified the term at the beginning of the interview. Following this, the interviewees were asked what obstacles they knew with regard to AI adoption, with the aim of bringing the problems back to mind. The following question then aimed to ask exploratively about possible measures that are necessary to enable physicians to make an informed adoption decision regarding AI applications (eg, training of physicians). If no further measures could be named, the measures already known from the literature or previous interviews were evaluated and concretized by asking specific questions. In total, we adjusted the interview transcript 5 times and pretested it twice to make sure that the questions were understandable and that the questions helped us to answer the overall research question.

### Data Analysis

We used an inductive approach to analyze the final set of 19 papers, following the methodologies outlined by Bandara et al [33]. After careful reading of the full texts of these papers, we extracted 117 relevant paragraphs that provided evidence for measures of AI adoption among physicians. For enhanced validity, 2 authors performed a 2-stage blind card-sorting allocation of measures to specific topics and levels. In the first stage, an author initially assigned each extracted paragraph to a preliminary set of topics and abstraction levels based on its content. In the second stage, a second author independently reviewed and sorted the same paragraphs, commenting on paraphrases, highlighting differences, and documenting areas of ambiguity. A key challenge in this process was that some measures could belong to multiple categories, while others required refinement to align their level of abstraction. For example, measures such as “in-house personalized learning programs” and “tools to be able to train” initially appeared as separate items but were later synthesized into the broader category “Enabling Practical Experience” to maintain conceptual clarity. To smooth the overall sorting outcome, the authors iteratively discussed the specific topics, their definitions, and measures’ abstraction levels until they reached convergence, which the literature refers to as constant comparison method [34]. We evaluated high-level measures through interviews, using targeted questions to concretize and refine the proposed measures. The interview findings helped concretize abstract measures and ensure that the identified themes were both relevant and applicable in practice.

By integrating insights from both the literature and the interviews, we identified 8 overarching measures that facilitate informed adoption decisions regarding AI applications by physicians. For instance, measures such as “in-house personalized learning programs for health care professionals” [35] and “tools to be able to train” [36] were synthesized into broader categories like Enabling Practical Experience.

### Ethical Considerations

The Ethics Committee of the University of Bayreuth approved our research proposal (approval 23-044) and checked if it was compliant with the General Data Protection Regulation. Before the interviews, all participants received a declaration of consent and information clearly stating that recordings would be used exclusively for research purposes and that all personal data would be pseudonymized and treated with strict confidentiality. Unique pseudonyms were used instead of names, and the data were securely encrypted. Participation in the interviews was completely voluntary and without any compensation, and participants could withdraw at any time without any negative consequences.

## Results

**Figure 1.**
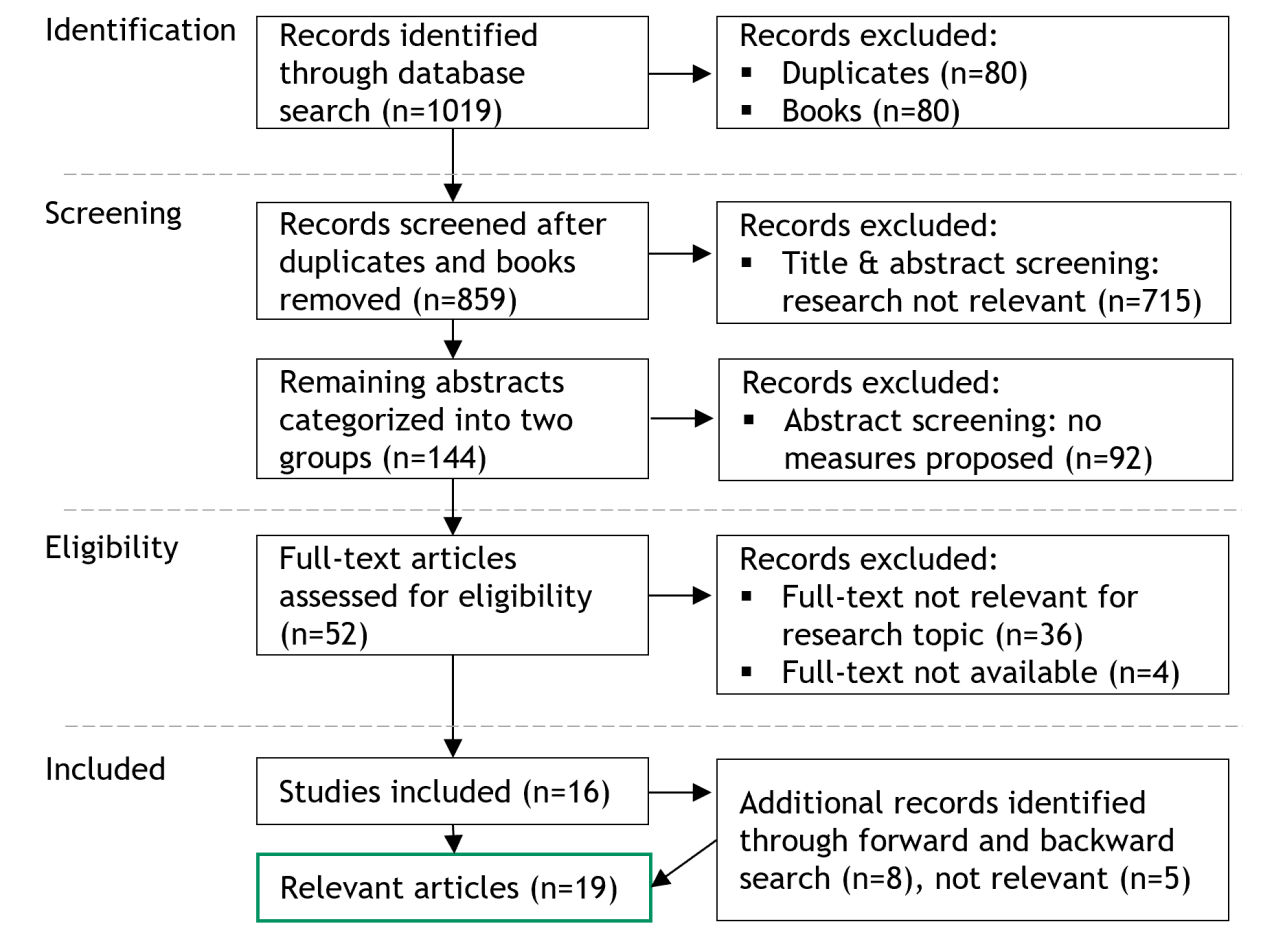
Search strategy.

As mentioned, a total of 19 articles formed the basis for our analysis of medical imaging diagnostics (Figure 1).

### Descriptive Results and Study Population

We interviewed 14 experts from Europe and Australia between March and May 2024. The experts are divided into 2 groups. In the first group, radiologists, potential medical users of AI applications in imaging diagnosis, were interviewed (n=5). Their insights help understand the practical challenges and needs regarding AI applications adoption in clinical workflows. In the second group, AI experts who have experience in working with physicians were interviewed. Their expertise offers insights into the design, development, and implementation challenges of AI in health care. The AI experts were composed of AI researchers (n=7) and developers of AI applications (n=2). We aimed to include a diverse range of participants, varying in age and the levels of experience of the interviewees. The “experience” metric refers to the number of years the participants have engaged with AI. The interviewees were initially contacted via mail, phone, or the authors’ professional social network and received a call if they had not replied to the initial request. The interviews were conducted face-to-face, either in person or virtually via a meeting platform, to reach trustworthy surroundings for the interview. In addition, the interviews were audio-recorded if the interviewees agreed and were subsequently anonymized for the article. See Table 2 for more information on the interview partners and the interview durations.

Derived from the literature and expanded and refined through the expert interviews, this section describes 11 measures of enablement, of which 9 can be summarized as Enabling Adoption Decision Measures (educating physicians, preparing future physicians, practically training physicians, integrating physicians in technology development, providing transparency, showing medical value, showing business value, establishing central expert panels, and establishing cross-disciplinary teams) and 2 as Supporting Adoption Measures (providing a marketplace for AI applications and providing implementation guidelines). Enabling Adoption Decision Measures includes measures aimed at empowering physicians to make informed decisions about the sensible adoption of AI applications in medical imaging diagnostics. Supporting Adoption Measures comprise measures aimed at ensuring that the physician receives support in the adoption process if a positive adoption decision is made.

**Table 2.** Descriptive characteristics of the participants and the data collection.

Participant number	Age (years)	Profession	Experience (years)	Interview duration (min)
E1	30	AI^a^ expert	3	53
E2	42	AI expert	7	30
E3	43	AI expert	6	29
E4	41	AI expert	6	18
E5	44	AI expert	6	43
E6	38	AI expert	4	54
E7	45	AI expert	5	50
E8	39	Radiologist	3	38
E9	41	Radiologist	3	66
E10	42	Radiologist	5	41
E11	41	Radiologist	2	38
E12	32	AI expert	5	50
E13	57	Physicist	10	47
E14	30	AI Expert	5	58
Average	40	—^b^	—	44

a AI: artificial intelligence.

b Not applicable.

### Enabling Adoption Decision Measures

#### Educating Physicians

Educating physicians about AI is emphasized in the literature and reinforced by the interviewees, making it a key element in enabling physicians to adopt AI. Many physicians are apprehensive about AI technology, as there are different levels of knowledge. Physicians feel pressure from their environment to deal with AI applications due to the hype the technology is currently experiencing in all domains (Expert 12), which, besides others, stems from AI applications going beyond human capabilities, which is difficult for people to understand (Expert 13).

To close the knowledge gap and mitigate concerns, Expert 7 emphasizes the need to familiarize physicians with the general topic of AI. Radiologists should be familiarized with AI terminology and hierarchy and clearly understand the diverse possibilities and challenges of AI applications [37]. To educate physicians and to increase trust in AI applications, targeted marketing campaigns with customized messages should be set up [38]. In addition, virtual social interactions can effectively raise awareness of AI applications in health care [39], and brochures explaining the benefits of AI applications should be made available in hospitals [40]. Face-to-face conversations play a crucial role in promoting AI understanding, as evidenced by Expert 2’s experience with workshops and lectures.

Besides familiarizing physicians with AI in general, Expert 12 suggests educating physicians about AI applications for their specific use case under consideration. AI cannot be viewed in a standardized way but must be considered separately for each domain and each use case (Expert 14). It is difficult to talk about AI in general terms as each use case potentially needs a different AI application. A deeper understanding of AI technology for specific areas of application will help to allay physicians’ concerns about its complete replacement. In this context, the experts agree that those who use AI applications will be economically successful, while not using AI applications will ultimately lead to losses (eg, Experts 7, 9, and 13).

Besides active measures, the adoption of AI applications is, according to Expert 10, a gradual process in which people will be familiarized with the technology slowly and often unnoticed. Eventually, AI applications will be steadily integrated into everyday life until they become an integral part of it.

#### Preparing Future Physicians

Besides training current physicians, it is essential to integrate AI topics into medical studies to optimally prepare future physicians for the challenges and opportunities coming up (Expert 4). The modernized curriculum should not only cover the basics of AI technology and its adoption in health care but also integrate ethical aspects and data science [35]. The inclusion of health informatics, computer science, and statistics in the study program is essential to provide students with the necessary technical skills for the effective use of AI applications and develop a critical understanding of ethical decision-making in AI-assisted medicine [37].

In addition, extended learning opportunities such as internships and electives in data science should promote interest in AI applications and digital health [41]. Expert 8 emphasizes the importance of involving medical students in AI topics at an early stage and networking with technology experts. The question is raised as to whether the upcoming physicians will be able to work without AI applications anymore, as there could be too much dependence on AI technology, having learned medical practice only with AI applications. However, Expert 10 mitigates these concerns by pointing out that the basic skills will remain while the role of the radiologist will evolve from pure image interpretation to a deeper understanding of technological processes. Expert 12 adds that change through new technologies always leads to adjustments in professional profiles, which is not a new phenomenon. Expert 2 sees the generational shift in the development of an addicted generation that will be inherently more skilled in a business context due to their early exposure to new technologies such as AI.

#### Practically Training Physicians

Enabling practical experience with AI applications is just as important as theoretical knowledge transfer to prepare physicians to make an informed adoption decision regarding AI applications in clinical practice. Physicians should be specifically trained in the use of AI applications for their respective tasks to be able to set up these systems effectively and interpret the generated results correctly [36]. Personalized learning programs and workshops with experts deepen the practical understanding of AI applications [35]. In addition, the use of third-party web tools, self-directed learning, and customized training programs enables physicians not just to make an adoption decision but also to use AI technologies effectively in the clinical setting and expand their practical experience [42]. Similarly, Expert 9 highlights that physicians can build trust through the practical application of AI applications. Further supporting the importance of skills development, Expert 7 points out that learning programming languages such as Python is essential to developing innovative medical methods.

#### Integrating Physicians in Technology Development

Koutsouleris et al [43] and Ganapathi and Duggal [41] emphasize that by physicians’ early involvement in the development process, AI applications can be tailored to effectively support medical tasks. This not only promotes the creation of practical, relevant, and efficient applications but also contributes to improving patient care. Expert 4 emphasizes the importance of involving clinicians early in the AI applications development process, stating:

*One reason why AI has not yet been more widely adopted is that it is not effectively integrated into clinical workflows. A key aspect of decision support is that the most relevant information needs to be presented to the clinician at the right time in the workflow*.

[Expert 4]

Expert 13 emphasizes that early integration promotes interdisciplinary collaboration between physicians and technicians, allowing radiologists to directly contribute to their specific needs and jointly optimize the development of AI applications. This not only improves the technical implementation but also promotes a deeper understanding and better communication between the disciplines involved. However, Expert 9 warns that physicians involved in the development of AI applications could often also pursue commercial interests, which could potentially lead to a distortion of development goals.

#### Providing Transparency

Transparency includes not only transparency with regard to the decision-making process of the AI algorithm but also transparency in the development of the AI application regarding, for example, which data was used to train the algorithm.

Regarding transparency in the algorithm, explainable AI**,** which allows users to follow the decision paths and how they are derived, can be a potential approach. Explainable AI can enable physicians to receive comprehensible and clinically meaningful explanations for the results of AI applications [44]. The ability to interpret decisions based on AI applications increases confidence in the tools used and helps physicians make informed judgments [45]. Transparency can be enhanced by visualizing decision paths [46].

Besides, AI applications should communicate when a prediction is uncertain and is likely to be unreliable, similar to an alert mechanism. Such an approach can increase the perceived reliability and trustworthiness of AI applications [47]. Expert 3 confirms that more transparency would help to create more trust:

The accuracy transparency would probably help a lot there. You know if we really trusted these algorithms to generalize well and they worked really, really well I could be really transparent about what they were doing where they were going to fail. We’d have a lot more trust in them.

[Expert 3]

Given the advantages of more transparency, Expert 11 appreciates it as it helps physicians to better understand decisions, while Experts 5 and 13 question the need for full explainability and compare it to implicit trust in human specialists. Expert 13 points out that there is already trust in the abilities of experts, even without their decision-making processes being fully transparent. The expert rhetorically asks where the difference lies with AI, especially in cases where life and death decisions are based on the experience of different physicians who vary in their level of experience. Expert 8 believes that with the standardization and testing of AI, the dependence on its explainability decreases, and trust based on performance and reliability increases.

#### Showing Medical Value

Detailed study reports provide physicians with comprehensive information on the development, performance, and limitations of specific AI models [48]. Through such reports, which include methodology, training, datasets, and bias aspects, among others, physicians can better assess the reliability and suitability of AI applications [48]. This need is confirmed by Expert 1, who emphasizes that physicians need a reference to better understand the value of using AI applications. Expert 5 underlines the importance of clinical trials to demonstrate the effectiveness of AI algorithms, explaining that it is not enough to look only at the “accuracy” parameter of AI applications but also at how valuable the integration of the AI application will be. Expanding on this view, Expert 13 emphasizes the need for high-quality studies on large networks. Interviewee 8 expresses critical concerns about the quality of studies, highlighting the need for independent research not commissioned by large companies to increase physicians’ confidence in AI applications. Expert 12 explains:

*AI should not be used as an end in itself, but to solve problems or improve health care. The value is created where there is the greatest benefit […]. In my opinion, there is no need for a physician to use AI; the need is for a physician to treat patients and provide them with good care - that’s the real need*.

[Expert 12]

Expert 11 adds:

*It’s about making things better for patients, improving diagnostic reliability and ensuring that it’s not just a danger. If you actively show that this is above all a great opportunity, you can break down many of these prejudices*.

[Expert 11]

#### Showing Business Value

Besides showing the medical value of AI applications, the business value due to time savings or additional income also has to be demonstrated (Expert 9). The need to clearly show radiologists how much time and money they can save by using AI applications is emphasized by Expert 8. In addition, it should be made clear which tasks AI applications offer added value for, as they are not equally suitable for all tasks. Expert 9 adds:

*[The radiologists] don’t even know whether this story is actually worthwhile. [...] Ultimately, the AI tools must actually bring us benefits [...] without us suffering financial losses.*

[Expert 9]

This skepticism often stems from past experiences where purchased solutions brought only costs and additional work.

The financial incentives of AI applications are further elaborated by Expert 10, who explains that many physicians will only use AI applications if they recognize a clear financial benefit. The expert states:

*Yeah, if I prove that it reduced my length of stay by 24 hours, then as a hospital I can calculate […] and multiply it by all patients for a year. That’s a bunch of new beds available, and I can bring new patients and increase my revenue, for example. But if I’m not able to demonstrate that, it’s not going to change my investment decisions.*

[Expert 10]

Expert 7 estimates the price of AI applications to be significantly lower compared to a second opinion from a colleague. Highlighting a further level of benefit, Expert 12 states that AI applications will only be used if they align with the corporate or practice strategy.

The importance of developing an appropriate payment system that rewards the use of AI applications in medical practice is pointed out by Expert 11. Expert 7 suggests introducing a billing figure for the use of AI applications, although it might take some time politically for such a measure to be implemented. The expert calls for politicians to focus more on AI applications in their considerations, stating, “So you have to expect and demand that politicians put AI at the centre of their considerations, because AI can prevent a lot of unelected work and work of equal value, repetitive work.”

#### Establishing Central Expert Panels

The importance of role models in medicine, especially involving experienced physicians who work directly with patients, is crucial for the successful adoption of AI applications. These role models can make a significant contribution to strengthening acceptance and trust in AI applications [41]. In order to provide a structured environment for discussions and policy development, it is recommended that task force committees be set up as central contact points for all AI-related topics [36]. Expert 7 states that such role models must come from the same discipline, as physicians tend to believe the testimonials of colleagues in their field rather than those of technology experts or consultants. Expert 11 adds that role models are necessary not only across disciplines but also for each individual specialty and each specific use case in order to meet the respective needs and approaches. Expert 9 points out the financial challenges associated with the creation of such an institution, which must ultimately be integrated into the health care system and financed.

#### Establishing Cross-Disciplinary Teams

Literature also suggests establishing interdisciplinary teams by bringing together medical doctors and specialists such as physicists, mathematicians, engineers, computer scientists, and biomedical researchers [48]. Besides, clinical domain specialists and data scientists, key stakeholders, and members from underrepresented populations, different genders, geographical regions, and racial groups should be integrated [49]. Investments must be made to build up multidisciplinary training programs from studentship to fellowship for clinical and non-clinical researchers [48]. These interdisciplinary teams are helpful in building a successful ML model as different specialists bring in knowledge that contributes to building an effective and efficient algorithm [50]. Further, bringing together different specialists helps in reducing biases [42,51]. In addition, the need for a national collaborative program between academia and industry dedicated to funding, developing, and mentoring AI leaders in radiology is underlined. These programs are designed to create a sustainable leadership base, complete with specialized training, structured mentoring, targeted funding, career networking, and sponsorship [52].

### Adoption Supporting Measures

#### Providing a Marketplace for AI Applications

Expert 8 and Expert 10 discuss the importance and structure of marketplaces for AI applications in health care that provide direct access to different products. Expert 8 states that such a marketplace can be very helpful, especially for newcomers to the industry, by providing a clear overview where you can see all available AI applications and their certifications at a glance. Expert 10 compares this approach to well-known online marketplaces such as Amazon and describes the platform as a kind of middleman that offers a portfolio of different products. However, the expert critically notes that these applications are not yet ideal and that there is no guarantee that all products are listed, which indicates that there is still room for improvement and further development in the quality and reliability of the AI applications on offer.

#### Providing Implementation Guidelines

The adoption of AI applications in the medical sector requires clear guidelines and protocols to provide physicians with guidance and safety when using these technologies. Fisher and Rosella [42] underline the importance of practical guidelines for the assessment and implementation of AI applications. These guidelines should provide physicians with a clear protocol documenting all steps and AI-based decisions to ensure guided and safe use. Nazer et al [49] support this view and point out that reporting guidelines during the early implementation phase of decision support systems are a step in the right direction. Expert 3 states that to solve some of these challenges, a stronger focus should be placed on implementation rather than focusing solely on the technology itself.

## Discussion

### Principal Findings

The derived measures can be divided into two categories: (1) Enabling Adoption Decision Measures, which includes measures (educating physicians, preparing future physicians, practically training physicians, integrating physicians in technology development, providing transparency, showing medical value, showing business value, establishing central expert panels, and establishing cross-disciplinary teams) aimed at enabling physicians to make informed decisions about the adoption of AI applications in medical imaging diagnostics and (2) Supporting Adoption Measures, which comprises measures (providing a marketplace for AI applications and providing implementation guidelines) aimed at ensuring that the physician receives support in the adoption process following a positive adoption decision.

In this section, the most prominent results of the literature review and the interviews are discussed. By exemplarily assigning the identified measures, which at the same time represent practical implications, to the factors influencing the adoption of AI applications in medical imaging diagnostics, we contribute to the adoption research stream (see Table 3).

Taking a closer look at the 9 enabling adoption decision measures in relation to the presented influencing factors in the Enabling Adoption Decision Measures subsection, the following connections can be found. The measures to educate physicians, prepare future physicians, and practically train physicians are directed to address AI literacy, system understanding, self-efficacy, perceived threat, technology receptiveness, and trust; for example, targeted in-house training programs and AI workshops allow physicians to test AI tools in simulated environments before clinical implementation, thus improving their AI literacy and affecting their self-efficacy. Furthermore, increasing knowledge will mitigate physicians’ concerns and fears [53]. Having knowledge about the specific AI application and its abilities will help to understand that AI applications are mainly focused on one specific task and are not directed to replacing a whole profession. In addition, physicians’ concerns about control loss and loss of autonomy can be reduced because physicians learn to deal with AI applications and get to know AI as a partner rather than a threat. In this regard, the general assumption is that only those physicians who do not use AI applications will be replaced by those who do (eg, Experts 7, 9, and 13).

Increasing knowledge and literacy will also increase physicians’ trust, as uncertainties will be reduced by giving users the transparency and competence they need to use AI applications safely and effectively [54]. The influencing factor “Trust” can further be addressed by the following measures: integrating physicians in technology development, establishing central expert panels, establishing cross-disciplinary teams, and providing transparency. For example, trust in AI technology can be increased by integrating physicians into the development of AI applications at an early stage, thereby bringing them together in cross-disciplinary teams and giving them a better understanding of how the technology works. This early involvement in multidisciplinary teams allows physicians to contribute their expertise and practical needs to the development process, resulting in a more user-friendly and practical application [55], thereby addressing the influencing factors “Burden” and “Workflow integration.”

The establishment of expert panels can also strengthen trust. The establishment of central expert panels can be attributed to the influencing factor “Social influence.” However, it is particularly important that these experts come from the respective specific medical disciplines (Expert 7). Through positive experiences and successes, these experts can inspire and motivate their colleagues to use AI applications themselves. In addition, providing more transparency, for example, through explainable AI or detailed study reports on the specific AI application, can further promote trust.

**Table 3.** Measures addressing the influencing factors (Hua et al [12]).

Enabling measures	Influencing factors											
	Burden	Value proposition	Perceived threat	Trust	Self-efficacy	AI^a^ literacy	System understanding	Technology receptiveness	Workflow integration	Ethicality	Social influence	Organizational readiness
Educating physicians			✓	✓	✓	✓	✓	✓				
Preparing future physicians			✓	✓	✓	✓	✓	✓				
Practically training physicians			✓	✓	✓	✓	✓	✓				
Integrating physicians in technology development	✓			✓					✓			
Providing transparency				✓								
Showing medical value		✓										
Showing business value		✓										
Establishing central expert panels				✓							✓	
Establishing cross-disciplinary teams				✓							✓	
Providing a marketplace for AI applications					✓							
Providing implementation guidelines					✓							

a AI: artificial intelligence.

Showing added medical value and showing added business value, on the other hand, are focused purely on the fact that physicians must know what benefits they can expect from the use of AI applications before making the adoption decision. The added value can be divided into 3 areas: more quality of care and time savings, which define medical value, and achieving more revenue, which defines business value. Improved quality means more accurate diagnoses, leading to better patient outcomes. Time savings can be reached by faster processing times of AI applications [56], allowing health care providers to see more patients or spend more time with each patient [13], thus also affecting revenue as higher patient throughput rates can be reached (Expert 10). In addition, the value of the technology needs to be evident not only in theoretical or laboratory settings but also when integrated into the workflow (Expert 4). In order to communicate the added value, literature [48] and interviewees (eg, Expert 1) suggest conducting detailed study reports for each specific AI application. It is particularly relevant that these reports exist for each specific use case, as AI applications come along with different added value for each use case, or possibly none at all (Expert 12). By describing, for example, the diagnostic accuracy of the AI application and the processing time of the regarded system in specific reports, the influencing factor ”Value proposition” can be addressed.

The importance of demonstrating the added value is further supported by the interviewees (eg, Experts 1, 8, and 10), who claim that the added value of AI applications could be more important in the adoption decision than solving the transparency problem of AI algorithms. Expert 7 illustrates this by stating that physicians already place trust in colleagues when consulting them for diagnostic suggestions without knowing exactly how they arrived at their recommendation.

If the adoption decision is positive, further measures are needed to support the physicians in actually adopting the specific AI application. The literature and interviews revealed 2 main measures, namely, providing a marketplace for AI applications and providing implementation guidelines. The 2 measures are targeted to give the physicians specific guidelines on how to choose and implement the specific AI application. Knowing about this support might also influence physicians’ self-efficacy*,* which will affect physicians’ adoption decisions.

In considering the influencing factors of Hua et al [12], only the factors of “Ethicality” and “Organizational readiness” cannot be addressed by the identified measures. The physicians’ perception that data protection guidelines are being adhered to (Ethicality) and that the IT infrastructure is sufficient (Organizational readiness) cannot be directly influenced by the measures targeted at enabling the user. Here, measures must be aimed at organizational level and not the user. Similarly, the responsibility for ethicality lies more with the technology developers, who must ensure that regulations are adhered to, and with the legislators, who may need to adapt or reinforce regulations.

Furthermore, the revealed measures suggest that a long-term perspective needs to be taken to achieve a continuous use of AI applications. Continuous usage refers to the sustained use of technology by adopters over an extended period following their initial acceptance decision [57]. For example, experts underline the importance of integrating AI topics into medical studies so that future physicians develop AI skills. This long-term view is crucial to ensure that AI applications are not just adopted initially but used permanently, as this is crucial for the sustainable life of information technologies [58]. This can be achieved through continuous training, regular adaptation of AI applications to new medical findings, and constant evaluation of implementation. By identifying measures aimed at promoting long-term usage, we underscore the importance of adopting research that focuses on continuous usage. To date, research on technology adoption in the health care context has primarily concentrated on explaining initial acceptance, with only a few studies emphasizing sustained usage [59].

By identifying specific enabling measures and mapping them to the factors known from the literature to influence the adoption of AI applications in medical imaging diagnostics [12], we contribute to the technology adoption research stream. While adoption research typically focuses on identifying facilitating and inhibiting factors in technology adoption, we extend this research by providing concrete, practical implications through the identification of specific measures. Our findings provide a detailed roadmap for practitioners and policymakers on how to effectively promote the adoption of AI among physicians by enabling them to make an informed adoption decision. To support real-world implementation, these measures can serve as guidelines for designing targeted policies, training programs, and institutional frameworks that facilitate AI integration in clinical practice. This practical approach bridges the gap between theoretical understanding and practical application, ensuring that the potential benefits of AI applications in medical imaging diagnostics are fully realized. By highlighting the role of organizational and regulatory support, we also emphasize the multifaceted nature of technology adoption and underscore the need for a holistic strategy that encompasses both individual and systemic interventions. This comprehensive perspective not only advances the academic discourse on technology adoption but also provides actionable insights for stakeholders seeking to improve the adoption of AI applications in health care.

### Limitations and Future Research

Despite the comprehensive approach and significant insights gained, this study has several limitations that need to be discussed. First, the sample size for expert interviews, while providing valuable qualitative data, follows convenience sampling and is focused on specific regions (Europe and Australia). This may limit the findings to specific health care systems and cultural contexts. Future research should aim to include a broader and more diverse set of participants from various geographical locations to validate and extend the applicability of the identified measures. Second, the findings rely on self-reported data from interviews, which may be subject to biases such as social desirability or recall bias. Triangulating these findings with quantitative data, such as surveys or usage metrics, could strengthen the validity of the conclusions. In addition, more experimental studies testing the effectiveness of specific enabling measures would help establish causal relationships and better inform practical implementation. Third, the scope of this study is primarily the user perspective, particularly that of physicians. However, the successful adoption of AI applications in medical imaging also depends on other stakeholders, such as medical imaging nurses, patients, and administrative staff.

Future research could focus on quantifying the relative impact of the identified barriers—technological, organizational, regulatory, and user-related—to better understand which factors most significantly hinder AI adoption in medical imaging diagnostics. Furthermore, the technological and organizational readiness factors, which our identified measures are not able to address, highlight the need for systemic changes that go beyond individual physician enablement. Future research should explore measures that must be taken in these areas of readiness to drive the adoption of AI applications in medical imaging diagnostics from technological, organizational, and macroeconomic perspectives. Examining organizational strategies, policy frameworks, and infrastructure investments is necessary to support AI adoption. Furthermore, comparative studies between institutions that have successfully adopted AI applications and those that have struggled could reveal further measures and best practices. In addition, longitudinal studies could provide valuable insights into the long-term effectiveness of the proposed measures in facilitating AI adoption. Research evaluating how these measures impact physician attitudes, trust, and practical AI usage over time would help refine implementation strategies. Furthermore, targeted studies on specific organizational barriers—such as interoperability challenges, workflow integration, and funding models—could offer more precise recommendations for health care institutions and policymakers.

### Conclusions

AI applications are considered to be promising in improving medical imaging diagnostics, as demonstrated by various research projects. However, previous research already pointed out various obstacles hindering the adoption of AI applications from an individual user perspective. Thus, we conducted a structured literature review and 14 semistructured expert interviews to identify measures enabling physicians to make an informed adoption decision. In total, we derived 9 enabling adoption decision measures and 2 supporting adoption measures, with the former empowering physicians by directly enhancing their AI knowledge, hands-on experience, and trust in AI applications, while the latter focusing on providing the necessary support, such as implementation guidelines and a marketplace for AI products, to facilitate adoption.

Measures aimed at building AI knowledge, enabling hands-on experience, and demonstrating the added value of AI applications appear particularly relevant to address physicians’ concerns and enable them to make informed decisions about adoption. By mapping specific measures to known influencing factors, our research provides a practical roadmap for practitioners and policymakers. This approach bridges the gap between theoretical understanding and practical application, helping to realize the full potential benefits of AI in medical imaging diagnostics. Furthermore, the methodology and findings of this study can serve as a model for similar inquiries in other medical fields, where user-centered adoption strategies are equally critical. By offering concrete, actionable recommendations, this study not only provides a framework for advancing the adoption of AI in medical imaging diagnostics but also lays the groundwork for transferable insights applicable across diverse medical domains. However, we also point out that focusing solely on the user perspective will not be enough to enable the successful adoption of AI applications. Instead, it is crucial to address technological, organizational, and macroeconomic barriers in equal measure. A holistic approach is therefore essential in order to effectively overcome the various obstacles to the implementation of AI applications.

If these barriers are successfully addressed, AI has the potential to significantly improve diagnostic accuracy, reduce physicians’ workload, and enhance patient outcomes through earlier detection and more precise treatment recommendations. Ensuring that physicians are adequately prepared to adopt AI is not only a matter of technological progress but ultimately a key step toward delivering higher-quality and more efficient patient care.

## Abbreviations

AI: artificial intelligence
IS: information systems
TAM: Technology Adoption Model
UTAUT: Unified Theory of Acceptance and Use of Technology

## References

[1] Kunduru AR (2023). Artificial intelligence advantages in cloud fintech application security. Cent Asian J Math Theory Comput Sci.

[2] Fridgen G, Halbrügge S, Körner MF, Michaelis A, Weibelzahl M Artificial intelligence in energy demand response: a taxonomy of input data requirements.

[3] Topol EJ (2019). High-performance medicine: the convergence of human and artificial intelligence. Nat Med.

[4] Hosny A, Parmar C, Quackenbush J, Schwartz LH, Aerts HJWL (2018). Artificial intelligence in radiology. Nat Rev Cancer.

[5] Killock D (2020). AI outperforms radiologists in mammographic screening. Nat Rev Clin Oncol.

[6] Bonekamp D, Kohl S, Wiesenfarth M (2018). Radiomic machine learning for characterization of prostate lesions with MRI: comparison to ADC values. Radiology.

[7] Chilamkurthy S, Ghosh R, Tanamala S (2018). Deep learning algorithms for detection of critical findings in head CT scans: a retrospective study. The Lancet.

[8] Buck C, Hennrich J, Kauffmann AL Artificial intelligence in radiology – a qualitative study on imaging specialists’ perspectives.

[9] Pagallo U, O’Sullivan S, Nevejans N (2024). The underuse of AI in the health sector: opportunity costs, success stories, risks and recommendations. Health Technol (Berl).

[10] Iyanna S, Kaur P, Ractham P, Talwar S, Najmul Islam AKM (2022). Digital transformation of healthcare sector. What is impeding adoption and continued usage of technology-driven innovations by end-users?. J Bus Res.

[11] Roppelt JS, Kanbach DK, Kraus S (2024). Artificial intelligence in healthcare institutions: a systematic literature review on influencing factors. Technol Soc.

[12] Hua D, Petrina N, Young N, Cho JG, Poon SK (2024). Understanding the factors influencing acceptability of AI in medical imaging domains among healthcare professionals: a scoping review. Artif Intell Med.

[13] Buck C, Doctor E, Hennrich J, Jöhnk J, Eymann T (2022). General practitioners’ attitudes toward artificial intelligence-enabled systems: interview study. J Med Internet Res.

[14] Venkatesh V, Morris MG, Davis GB, Davis FD (2003). User acceptance of information technology: toward a unified view. MIS Q.

[15] Davis FD (1989). Perceived usefulness, perceived ease of use, and user acceptance of information technology. MIS Q.

[16] Venkatesh V, Speier C, Morris MG (2002). User acceptance enablers in individual decision making about technology: toward an integrated model. Decision Sciences.

[17] Jussupow E, Spohrer K, Heinzl A, Gawlitza J (2021). Augmenting medical diagnosis decisions? An investigation into physicians’ decision-making process with artificial intelligence. Information Systems Research.

[18] Hennrich J, Fuhrmann H, Eymann T Accelerating the adoption of artificial intelligence technologies in radiology: a comprehensive overview on current obstacles.

[19] Gama F, Tyskbo D, Nygren J, Barlow J, Reed J, Svedberg P (2022). Implementation frameworks for artificial intelligence translation into health care practice: scoping review. J Med Internet Res.

[20] Marco-Ruiz L, Hernández MÁT, Ngo PD (2024). A multinational study on artificial intelligence adoption: clinical implementers’ perspectives. Int J Med Inform.

[21] Davis FD, Bagozzi RP, Warshaw PR (1989). User acceptance of computer technology: a comparison of two theoretical models. Manage Sci.

[22] Prakash AV, Das S (2021). Medical practitioner’s adoption of intelligent clinical diagnostic decision support systems: A mixed-methods study. Information & Management.

[23] Hsieh PJ (2015). Healthcare professionals’ use of health clouds: Integrating technology acceptance and status quo bias perspectives. Int J Med Inform.

[24] Venkatesh V, Sykes TA 'Just what the doctor ordered': a revised UTAUT for EMR system adoption and use by doctors.

[25] Hu PJ, Chau PYK, Sheng ORL, Tam KY (1999). Examining the Technology Acceptance Model using physician acceptance of telemedicine technology. J Manag Inf Syst.

[26] Hung SY, Ku YC, Chien JC (2012). Understanding physicians’ acceptance of the Medline system for practicing evidence-based medicine: a decomposed TPB model. Int J Med Inform.

[27] Benbasat I, Barki H, University of British Columbia, Canada, HEC Montréal (2007). Quo vadis TAM?. JAIS.

[28] Holden RJ, Karsh BT (2010). The Technology Acceptance Model: its past and its future in health care. J Biomed Inform.

[29] Weeger A, Gewald H (2015). Acceptance and use of electronic medical records: an exploratory study of hospital physicians’ salient beliefs about HIT systems. Health Syst (Basingstoke).

[30] Webster J, Watson RT (2002). Analyzing the past to prepare for the future: writing a literature review. MIS Q.

[31] Schultze U, Avital M (2011). Designing interviews to generate rich data for information systems research. Information and Organization.

[32] Myers MD (2019). Qualitative Research in Business and Management.

[33] Bandara W, Furtmueller E, Gorbacheva E, Miskon S, Beekhuyzen J (2015). Achieving rigor in literature reviews: insights from qualitative data analysis and tool-support. CAIS.

[34] Corbin JM, Strauss AL (2015). Basics of Qualitative Research: Techniques and Procedures for Developing Grounded Theory.

[35] Wiljer D, Salhia M, Dolatabadi E (2021). Accelerating the appropriate adoption of artificial intelligence in health care: protocol for a multistepped approach. JMIR Res Protoc.

[36] Nilsen P, Svedberg P, Nygren J, Frideros M, Johansson J, Schueller S (2022). Accelerating the impact of artificial intelligence in mental healthcare through implementation science. Implement Res Pract.

[37] Tang A, Tam R, Cadrin-Chênevert A (2018). Canadian Association of Radiologists white paper on artificial intelligence in radiology. Can Assoc Radiol J.

[38] Sebastian G, George A, Jackson G (2023). Persuading patients using rhetoric to improve artificial intelligence adoption: experimental study. J Med Internet Res.

[39] Ho MT, Le NTB, Mantello P, Ho MT, Ghotbi N (2023). Understanding the acceptance of emotional artificial intelligence in Japanese healthcare system: a cross-sectional survey of clinic visitors’ attitude. Technol Soc.

[40] Xiang Y, Zhao L, Liu Z (2020). Implementation of artificial intelligence in medicine: status analysis and development suggestions. Artif Intell Med.

[41] Ganapathi S, Duggal S (2023). Exploring the experiences and views of doctors working with artificial intelligence in English healthcare: a qualitative study. PLoS ONE.

[42] Fisher S, Rosella LC (2022). Priorities for successful use of artificial intelligence by public health organizations: a literature review. BMC Public Health.

[43] Koutsouleris N, Hauser TU, Skvortsova V, De Choudhury M (2022). From promise to practice: towards the realisation of AI-informed mental health care. Lancet Digit Health.

[44] Asan O, Bayrak AE, Choudhury A (2020). Artificial intelligence and human trust in healthcare: focus on clinicians. J Med Internet Res.

[45] Markus AF, Kors JA, Rijnbeek PR (2021). The role of explainability in creating trustworthy artificial intelligence for health care: a comprehensive survey of the terminology, design choices, and evaluation strategies. J Biomed Inform.

[46] Li Z, Wang L, Wu X (2023). Artificial intelligence in ophthalmology: the path to the real-world clinic. Cell Rep Med.

[47] Bates DW, Auerbach A, Schulam P, Wright A, Saria S (2020). Reporting and implementing interventions involving machine learning and artificial intelligence. Ann Intern Med.

[48] Gilbert FJ, Smye SW, Schönlieb CB (2020). Artificial intelligence in clinical imaging: a health system approach. Clin Radiol.

[49] Nazer LH, Zatarah R, Waldrip S (2023). Bias in artificial intelligence algorithms and recommendations for mitigation. PLOS Digit Health.

[50] Tice AM, Farag HA (2019). Machine learning in microbiology: finding the signal in the noise. Clin Microbiol Newsl.

[51] Raman R, Dasgupta D, Ramasamy K, George R, Mohan V, Ting D (2021). Using artificial intelligence for diabetic retinopathy screening: policy implications. Indian J Ophthalmol.

[52] Doo FX, McGinty GB (2023). Building diversity, equity, and inclusion within radiology artificial intelligence: representation matters, from data to the workforce. J Am Coll Radiol.

[53] Alsultan K (2023). Awareness of artificial intelligence in medical imaging among radiologists and radiologic technologists. Cureus.

[54] Malerbi FK, Nakayama LF, Gayle Dychiao R (2023). Digital education for the deployment of artificial intelligence in health care. J Med Internet Res.

[55] Huo W, Yuan X, Li X, Luo W, Xie J, Shi B (2023). Increasing acceptance of medical AI: the role of medical staff participation in AI development. Int J Med Inform.

[56] Hennrich J, Ritz E, Hofmann P, Urbach N (2024). Capturing artificial intelligence applications’ value proposition in healthcare - a qualitative research study. BMC Health Serv Res.

[57] Kim HW, Chan HC, Chan YP (2007). A balanced thinking–feelings model of information systems continuance. Int J Hum Comput Stud.

[58] Bhattacherjee A (2001). Understanding information systems continuance: an expectation-confirmation model. MIS Q.

[59] Abouzahra M, Guenter D, Tan J (2024). Exploring physicians’ continuous use of clinical decision support systems. Eur J Inf Syst.

